# How Long Is Too Long: An Individual Time-Window for Motor Planning

**DOI:** 10.3389/fnhum.2019.00238

**Published:** 2019-07-11

**Authors:** Anat Dahan, Rotem Bennet, Miriam Reiner

**Affiliations:** ^1^Department of Education in Science and Technology, Technion Israel Institute of Technology, Haifa, Israel; ^2^Department of Software Engineering, ORT Braude College of Engineering, Karmiel, Israel

**Keywords:** motor planning, individual differences, hippocampus, optimal control, planning interval

## Abstract

Human motor response time (RT) is determined by the matureness of the preceding neural motor planning process. In the current study, we characterize the temporal boundaries required for the motor planning process, and its impact on the overall motor RT. In particular, we contrasted short and long planning times by measuring the resulting differences in motor RTs, in an attempt to find whether an optimal planning time for minimal RT exists. Using a “Timed Response” paradigm, we presented participants with varying planning intervals prior to a requested motor response and studied their effect on the timing of initiation of the following movement. We found that, as expected, reaction time shortens as more planning time is provided, yet only until reaching a minimal RT, after which additional planning time increases the motor RT, thus creating a U-shaped behavioral function. Furthermore, since the minimal RT was found to be an individual characteristic, we suggest that there is an individual time window for motor planning.

## Introduction

Mechanisms of movement planning are a subject of much interest. A wide body of evidence exists showing neuronal activity anticipating specific movements before their onset (Li et al., 2015) and providing evidence and models for movement planning (Viviani and McCollum, 1983; Viviani and Schneider, 1991; Viviani and Flash, 1995).

In a previous experiment (Dahan and Reiner, 2017), we witnessed an interesting phenomenon: Using a “timed response” paradigm in a virtual-game-like environment, we presented participants with two planning intervals of 25 and 350 ms before motor response was required. We found that when planning time was insufficient, movement onset was delayed, and as the planning interval duration became further extended, movement onset time became shorter. We concluded that the shorter onset time was correlated with a maturity of the motor plan. We interpreted that the short time interval was insufficient for the motor plan to mature, and once available time was sufficient, motion onset became faster. In this study, we wanted to check what happens when planning time is further extended.

The “timed response” paradigm initially was introduced by Hening et al. (1988), where they studied the processes that take place as participants use information from a presented target to set the amplitude of an impulse of isometric elbow force. The participants were presented with separate cues to time initiation of movement and to display the target location. When the time between target presentation and movement initiation was less than 100 ms, the participants performed a default response, unrelated to the location of the target. In a subsequent experiment (Ghez et al., 1997), participants were required to move towards one of several choices of targets. They were instructed to initiate the movement at the last of a sequence of auditory cues. The target to reach was shown at various times before the final auditory Go cue. They measured the production of force at the different directions and found that when subjects were forced to make choices quickly (available planning time <100 ms), they moved towards the targets randomly when the targets were distant, and between the targets, if the target locations were close.

Kohen et al. (2017) extended this paradigm to an obstacle avoidance reaching task, in order to investigate movement planning of curved trajectories. They found that after short planning intervals (25 ms), the trajectories that followed were target specific, even at onset, but these trajectories were more variable and with a larger angular deviation from a straight line toward the target when compared to trajectories that followed a longer planning interval (350 ms). This result suggests that when planning time was not sufficient, participants performed a “sub-optimal trajectory” and applied improvements on the later parts of the movement “on the fly.” However, they found no effect of the two planning-time conditions on movement onset latencies. These findings are different than those in our previous study where we noticed a difference in onset times. A possible explanation for this difference is that in our experiment short and long conditions were mixed randomly within a block, and in the described experiment long and short conditions were presented in separate sessions, setting a different “mode of action” for each session.

Object reaching movements consist of two problems: choosing the target of the movement and defining a motor plan for the movement. Cisek (2007) introduced a model suggesting that these are not separate serial processes, where a target is selected and then its motor plan is prepared, but rather, are parallel processes that occur within the very same neural circuits. Accordingly, the actual actions we perform are viewed as a constant competition between different internal representations of the potential actions. The framework presented by Cisek and Kalaska (2005) is called the “affordance competition hypothesis.”

It was further found that when the reaching movement is unconstrained, planning time is mainly effected by target selection (Cisek and Kalaska, 2005). When the task involves constraints or obstacles, additional preparation time is required. Wong et al. (2016) assessed the response time (RT) cost of trajectory representation. One group of subjects was shown only a target and barriers, and a second group was cued to a path that avoided the barriers, which they were instructed to follow. They found kinematic similarities between groups. However, the group that had to plan the movement was 94.61 ms slower in initiation of movement compared with the group that was provided cues for the path.

A similar paradigm has been studied in monkeys (Riehle and Requin, 1989; Crammond and Kalaska, 2000), in instructed delay tasks, where an instruction cue was separated by a temporal delay from a movement initiation Go cue. The researchers recorded activity from hundreds of neurons including neurons in the dorsal premotor cortex and in the primary motor cortex. Findings showed that at a behavioral level, RT decreased and then plateaued as a function of the delay interval between the instruction and the Go cue. The reduction mainly occurred during the first 200 ms, suggesting that some time-consuming preparatory process is given a head start by the delay. However, at a neuronal level, there was a striking heterogeneity in firing rates between different cells. During the delay interval, some neurons increased their firing rate, some decreased it, some plateaued, and others undulated. Shenoy et al. (2011) highlighted the interesting contrast between the heterogeneity of neural responses at a cellular level, with the monotonic decrease in RT on the behavioral level. They examined how the neural activity during the first 200–300 ms of the delay period relates to the following RT. Accordingly, they introduced the “Optimal Subspace Hypothesis,” suggesting that for a desired movement there exists a subspace of neuron states that will produce it. Hence, the process that takes place during motor preparation is an optimization of the neurons’ firing configurations so the neurons firing is within this subspace, a process that can take place at different speeds and along different paths.

In our previous results, we provided participants with very short (25 ms) planning interval for a motor act, and with long durations of 350 ms. Our findings showed that very short preparation was correlated with a longer RT. Sufficient planning time was found to correlate with a shorter RT, and we assumed that the motor plan readiness level was higher when sufficient planning time was allowed. The question that came us was what happens when we further increase the preparation time? Will the readiness level still increase? and—how long is too long for increased motor readiness level? Stated differently, how long is too long for the participant to lose the memory of the specifics of the mature motor plan?

As it became clear in previous experiments (Reiner et al., 2014) that motor working memory (WM) is involved in motor learning and performance, as can be seen by theta rhythm that occurs in the human neocortex and hippocampus, in sleep and on waking (Diekelmann and Born, 2010; Reiner et al., 2014), which is often directly gated during the period of WM (Raghavachari et al., 2001). Motor execution and motor learning involve formation of motor memories, that decay over time (Ingram et al., 2013). WM can be defined as a set of functions that enable the temporary maintenance of information and the access to this information in order to perform higher-level cognitive operations. The half-life time duration of WM patterns is limited. For instance, iconic memory only lasts in WM about 500 ms and is subject to interference by newly incoming visual signals (Sperling, 1960). Here, we test the hypothesis that the readiness of a motor plan is held for a limited time, and then a decay process starts. If this is correct, the time needed for response when the preparation is short should be relatively long, decreasing when the preparation-readiness level is higher, and increasing again when the preparation readiness is so long that the pattern of WM decays. Thus, here we hypothesized that longer planning intervals would result in decay as can be seen in processes that involve WM (Peterson and Peterson, 1959), and should be reflected in a U shaped RT.

We further suggest that characterization of the personal timing pattern can serve as an indicator for the ability to create and obtain a motor plan, and can, therefore, assist in the medical surveillance of patients with neurological disorders.

## Materials and Methods

### Participants

The experiment was performed on 16 participants, with normal or corrected vison, no known motor problems, and no known diagnosis of attention deficit disorder, ages 22–45 (average = 32.1, SD = 6.9). Informed consent was received from all participants, and the study was approved by the Technion ethics committee.

### Apparatus

The experiment was performed using a combination of a projected (using a projector with a resolution of 1,280 × 720 with refresh rate of 120 Hz), ecologically valid, virtual environment and a robotic arm (also called Touch^TM^ X, manufactured by Geomagic, 2014). During the experiment, the participants looked downwards at a semi-transparent horizontal mirror (size 82 W × 55 H cm) and viewed the experimental setting. They controlled the movement by manipulating the pen-like stylus of a Phantom robotic arm, which was placed under the mirror surface. The Phantom and subject’s arms were both invisible to the subject, who saw the movement of the Phantom as movements of a pen-like device upon a surface. The robotic arm exerted forces on the participant’s arm, which created a feeling of natural movement over a physical surface with friction. It is composed of four links and three joints and is able to move in working space of size (16 W × 12 H × 12 D cm).

### Procedure and Task

Participants held a stylus at a fixation point and were trained to initiate a movement towards a target, only after the fourth of four consecutive tones, while avoiding obstacle points on the route.

**Figure 1:**
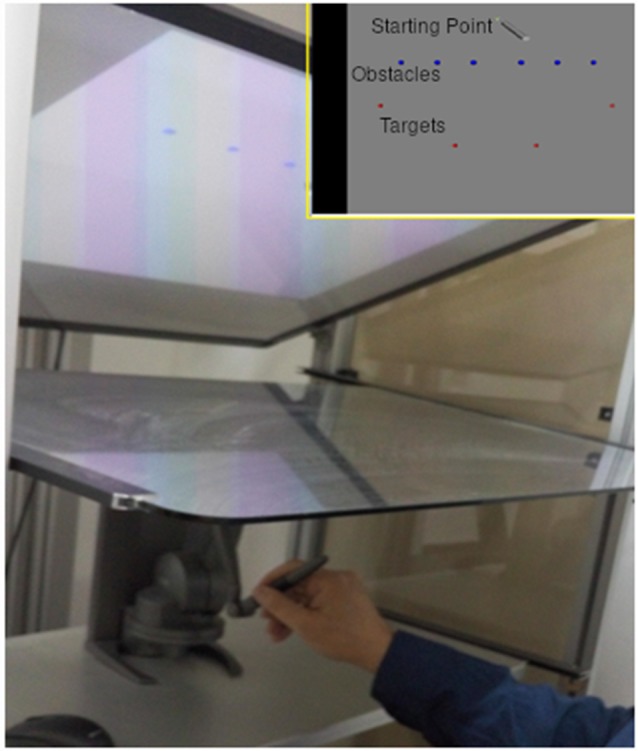
Experimental setting. Participants performed a reaching movement holding a pen like stylus while looking down at a semi-transparent mirror (main image). They performed the movement from starting point to one of the targets (red) while avoiding the obstacles (blue) on the way (top right image).

**Figure 2:**
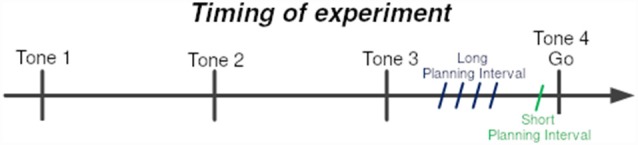
Timing of experiment. Subjects were trained to leave the starting point at the last of sequence of four tones at 700 ms intervals. The 4th tone constituted the Go signal. One of the four targets was presented prior to the Go signal by either the Short-planning interval of 25 ms or the Long-planning interval of 250, 350, 450, or 550 ms. The four Long-preparation intervals were presented in four different blocks of trials.

Obstacles were placed to force a non-trivial reaching movement. There were six obstacles between the starting point and the target location, set beside each other at stationary locations (see Figure 1). At each trial, one of four possible targets was selected randomly, and appeared at a fixed radius from the center, but at different spatial angles. The selected target appeared at one of two time intervals before the last tone (see Figure 2), short and long:

1. Short Planning Interval: target appeared 25 ms before the last tone.
2. Long Planning Interval: target appeared either 250/350/450/550 ms before the last tone.

### Design

The experiment included four different blocks corresponding to the four possible long planning intervals. The blocks were presented at a random order for each subject. To ensure the different blocks evaluated the same initiated motor planning process despite the different planning interval conditions, each block consisted of trials of the block’s Long planning interval condition (e.g., 250 ms) randomly mixed with Short planning interval trials of 25 ms, thus not allowing each block to induce a steady state of planning.

Each of the four targets appeared at random order, six times for each planning interval condition (Short/Long), resulting in 6 (replications) * 4 (targets) * 2 (planning intervals) = 48 trials, in each block. Each player performed up to 10 practice trials before the test, to get used to initiating movement at the fourth tone, and not before. The use of the robotic arm was intuitive and natural as holding a pen and all participants were able to perform the task.

### Data Analysis

Tracking data of the robotic arm movement in each trial was measured and recorded at a sampling rate of 120 Hz (48 trajectories for each block). Data were further analyzed using MATLAB (The Mathworks, Inc., Natick, MA, USA). Tracking data was smoothed using the sgolayfilt function (Savitzky and Golay, 1964). Tracking data was derived to calculate velocity. Onset of movement was calculated as the time when velocity passes a threshold (*v* > 0.0001 in game units) after the fourth tone, or as zero otherwise, since we only started recording after the fourth tone.

Starting movement recording only after the fourth tone is a limitation of this study, as fractions of movements that started before this tone they were not recorded. We performed a *post hoc* analysis of these trials. There were 423 out of a total of 2,800 that started before the fourth tone. As we generally found that participants started to move earlier after a long planning interval than after a short planning interval, we analyzed that if we had taken the times before the tone into account, we would have had more significant statistical values, as there were more Long planning interval trials that started before the 4th tone than Short planning interval trials (i.e., 359 vs. 64, respectively).

### Statistical Analysis

To test the effects of the different factors on the reaction time, we used a linear mixed-effects regression model, controlling for the between-subjects variability by defining the Subject factor as a random effect. In each analysis, subsets of the following variables were used as within-subject fixed effects: per-trial planning interval condition (Short/Long), per-block planning interval condition (between Long intervals—250/350/450/550), and per-trial planning interval condition (between all the planning intervals, Short and Long—25/250/350/450/550). A significance threshold of *p* < 0.05 was set for all the statistical analyses. *T*-tests were used for *post hoc* pairwise comparisons, with a Bonferroni-correction of the *α*-value were needed to account for multiple-comparisons familywise error rate (FWER). The data are reported as mean ± standard error of the mean (SEM). All statistical analysis was done using JMP, Statistical analysis^TM^ 13.0, from SAT, data analysis software. The Restricted Maximum Likelihood (REML) method was used for fitting the mixed-effects models, as it handles well also cases of unequal sample sizes (JMP, 2013).

## Results

### Delayed Movement Onset RT for Short vs. Long Motor Planning Interval Trials

**Figure 3:**
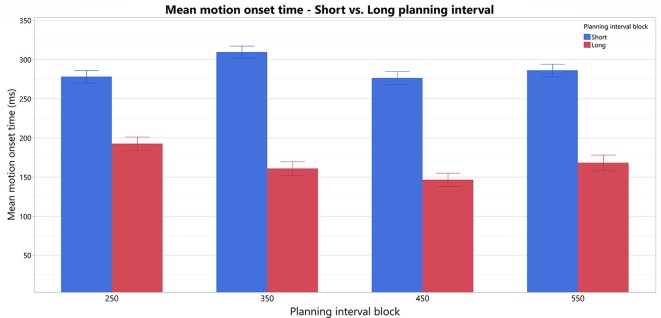
Comparison of motion-onset Start times in Short vs. Long planning-interval trials in each of the blocks. There is an overall significant difference between Short and Long planning-interval trials.

To test our prediction that trials with Short planning interval of 25 ms require significantly more additional motor preparation than longer planning interval trials, we analyzed the effect of a trial’s condition (Short/Long) on the RTs and its possible interactions with the block’s Long planning interval condition, while testing the possible effect of the specific target position (as a fixed effect) and controlling for the individual differences between the subjects (as a random effect). The fitted model found a significant effect of the trial’s Short/Long planning interval condition on the movement onset RT (*F*_(1,15)_ = 58.5, *p* < 0.0001), no main effect of the block’s Long planning interval (*F*_(3,45)_ = 1.26, *p* > 0.3) and a significant interaction of the trial condition with the block’s Long planning interval (*F*_(3,45)_ = 2.9, *p* < 0.05). Therefore, movement onset RT in the short trials, with planning interval of 25 ms (287.4 ± 21.3 ms), was found to be significantly higher than for the Long planning interval trials (166.9 ± 21.3 ms), for any of the long durations thus consistently indicating the need in motor planning beyond 25 ms (see Figure 3). *Post hoc* Tukey HSD analysis of the inetraction between the trial condition and the Long planning interval, found no significant effect of the block on the differences between the Short and Long planning durations. The main effect of the Target position was also significant (*F*_(3,45)_ = 3.4, *p* < 0.05), and Tukey HSD *post hoc* analysis found that the RT was significantly higher in trials with target 1 (leftmost, 240.9 ms) than in trials with target 3 (middle-right, 213.4 ms). The interaction between the Target and the trial’s condition was also significant (*F*_(3,45)_ = 3.4, *p* < 0.01), and Tukey HSD *post hoc* analysis found that the difference between target 1 and 3 is only in the Short trials, whereas there is no significant difference between RT’s for the Long trials in the four targets.

### U-Shape Window of Motor Planning

**Figure 4:**
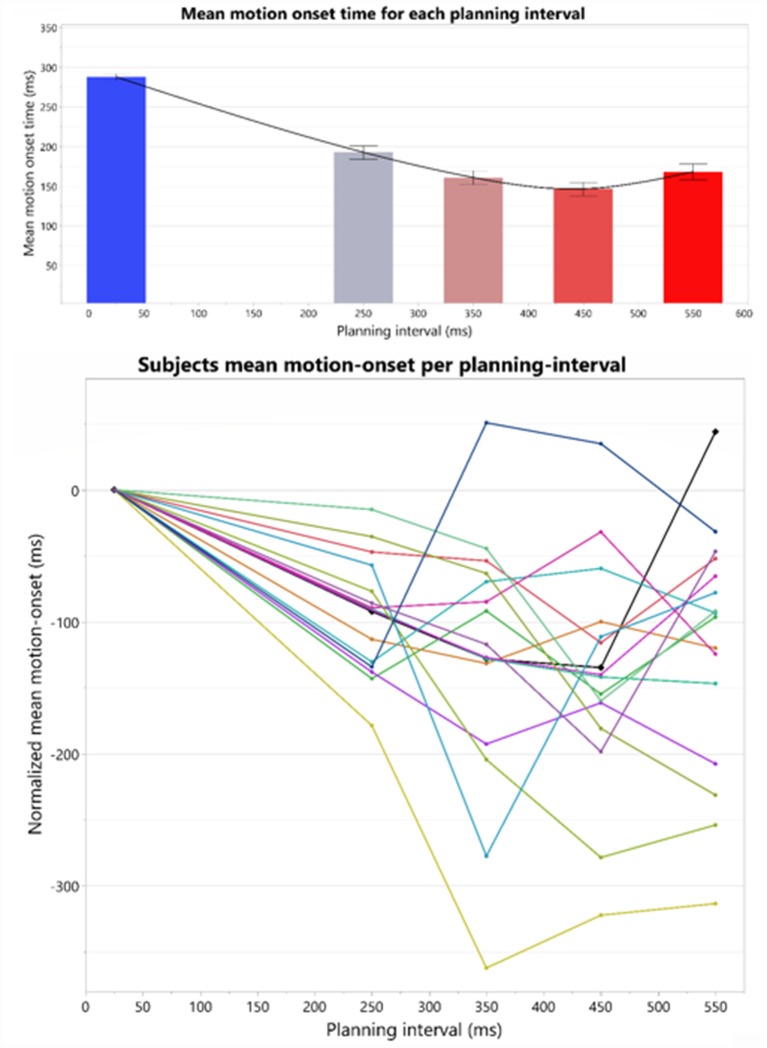
Top: overall U-shape pattern across all the subjects. The bars represent the means and standard-errors of motion-onset response times (RTs) in each motor planning interval condition, where the four right bars are calculated over the Long trial RTs in each of the blocks, and the leftmost bar is calculated over the Short trials RTs across all the blocks. Bottom: individual motion-onset RT for each subject and each planning interval condition, averaged over four targets, and all trials for each target. All graphs were drawn together and aligned to a 0 starting time, to see overall similarities and differences in response pattern.

We examined the average Reaction Time (RT) following each planning interval. The average RT’s revealed an interesting pattern: as planning interval becomes longer the RT does not simply reach a minimal plateau, but rather there seems to be an window of target presentation for a minimal RT, after which RT increases. This patterns of RT’s results in an overall U-shape as can be seen in Figure 4.

**Figure 5:**
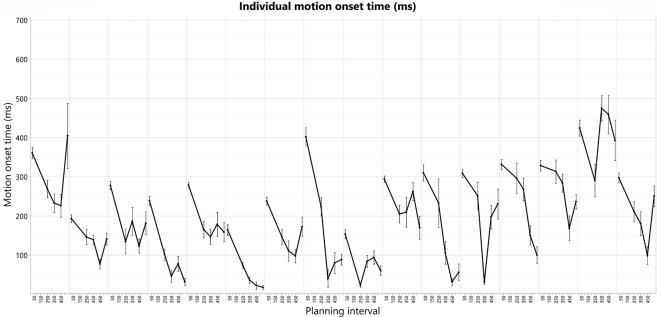
Individual motion-onset RT for each subject and each planning interval condition, averaged over four targets, and all trials for each target.

The individual patterns of motion-onset RTs highly vary between the subjects, as was evident in the consistently significant variability explained by the subjects’ factor in any of the aforementioned random-effects tests we ran, in which we treated these between-subjects patterns as random noise to control for, when testing within-subject effects (e.g., Short-Long trials difference) across all the subjects in each experimental group. The individual patterns of motion-onset RTs for each planning interval block, are shown in Figure 4 (bottom) and Figure 5. While a U-shaped pattern is evident for most participants, for two participants there seems to be only a downward part, possibly because they have not reached their individual minimal RT.

To test for the existence of a U-shape RT pattern across the subjects, and whether it has individually unique characteristics, we have extracted the U-shape properties separately for each subject. The extracted properties were the planning intervals and respective RTs of the U-shape parts—leftmost max mean RT (Max1—“too short” planning interval), the middle min mean RT (Min—minimal planning interval), and the rightmost mean max RT (Max2—“too long” planning interval). Extracting these parameters for each subject aligns the subjects’ U-shapes and enables testing for the pattern’s general statistical significance across the subjects while controlling for the remaining individual differences as a random factor. The “too short” planning interval point was trivially taken as the Short, 25 ms condition for all the subjects (which was shown above to have consistently longer RT). The individual U-shape minimum point was taken as the planning interval of the first local-minimum RT, and the individual U-shape “too long” point was taken as the planning interval of the following local-maximum RT; see Table 1.

**Table 1:** Average onset time for each participant in each planning interval.

Subject number	25	250	350	450	550
1	**360.610575**	268.3226	232.4907	**225.8243**	**404.56715**
2	**193.0131125**	145.8275	139.1611	**77.08025**	**140.8277**
3	**277.697225**	**134.57795**	**185.8259**	122.91175	181.24275
4	**238.948775**	100.8293	**46.24815**	**77.4969**	31.24875
5	**278.3222**	164.9934	**146.6608**	**178.3262**	158.327
6	**163.951775**	73.74705	35.8319	22.08245	**17.08265**
7	**237.5946625**	146.24415	110.41225	**97.4961**	**172.07645**
8	**401.9630875**	223.3244	**39.58175**	79.58015	**88.3298**
9	**153.4313625**	**22.91575**	83.74665	**93.74625**	59.9976
10	**294.0507375**	**204.57515**	209.1583	**262.07285**	169.57655
11	**309.8834375**	232.90735	105.41245	**31.24875**	**55.8311**
12	**308.4251625**	251.23995	**30.8321**	197.07545	**230.40745**
13	**331.3409125**	295.8215	267.90595	150.41065	**99.996**
14	**328.3202**	313.3208	283.73865	**168.3266**	**236.24055**
15	**424.2538625**	**289.9884**	**474.981**	459.1483	392.4843
16	**297.1756125**	211.24155	179.9928	**98.74605**	**250.40665**

*Selected Max1, Min and Max2 are marked in bold for each participant. Participants 6 and 13 have only the Max1 and Min points*.

To test the significance of the individual U-shape RT patterns, we ran a linear mixed-effects modeling analysis of the U-shape downwards and upwards segments. Our analysis tested for the significance of the within-subject differences between the RTs in trials of the three U-shape parts (Max1, Min, Max2) as found for each subject while controlling for the individual between-subject variability. For this analysis, two participants who did not have an upward part were excluded, remaining with *n* = 14 subjects. The test found a significant difference between the RTs of the U-shape parts (*F*_(2,27.4)_ = 33.7, *p* < 0.0001). *Post hoc* Tukey HSD analysis found that Min point RT (115.3 ± 25.4 ms) were significantly lower than Max1 (“too short”—25 ms) point RTs (293.1 ± 24.6 ms), as expected. Most critically, Min point RTs were also significantly lower than Max2 (“too long”) planning-interval point RTs (203.7 ± 25.4 ms), which in turn were also significantly lower than the Max1 RTs. Taken together, these differences show that there is a U-shape pattern characterizing the effect of the planning interval on the motion-onset RT, where there is not only “too little” time for planning, but apparently also a “too late” point in time where there is a significant degradation in the motor readiness.

## Discussion

We tested our hypothesized window of motor planning, during which any additional planning time first facilitates the maturity of the motor plan, thereby shortening the motion-onset RT, and then, when the time-window ends, decreases the motor readiness, which leads to an increase of the motion-onset RT. According to the existing research literature, the motion-onset RT is expected to decrease with additional planning time available, until reaching a plateau when the planning interval is enough for the completion of the motor plan (Riehle and Requin, 1989; Crammond and Kalaska, 2000; Shenoy et al., 2011). No significant increase in the RT was expected, due to over-planning in the scale of 250–550 ms studied here, and therefore, this particular hypothesis was set as the main conjectured novelty of our study. We found that 14 of 16 subjects had an increase in RT. It may be that other subjects have not yet reached their timing of minimal response. This should be further tested in a follow-up experiment with a longer range of planning intervals.

Shenoy et al. (2011) suggested an explanation for the additional planning time when the planning interval is too short, as a preparatory state needed for neuronal activation pattern to reach a state needed for the desired movement. They termed the state of all relevant neuron populations as an optimal subspace. They suggested that for different trials, the state may be reached from a different starting point, through a different path, and at a different rate. In this study, we see that while the pattern of a U-shaped RT occurred across subjects, we find here that each subject has her/his own individual planning interval. According to the optimal subspace hypothesis, this may be interpreted as individual characteristics of each individual neural population, which yield different paths, and rates.

These findings may also be interpreted in light of the “affordance competition hypothesis” (Cisek, 2007). When the planning intervals were short, the movements’ onsets were delayed. This may be interpreted as the time needed for the visual target stimuli to be attended, and for the data to be propagated to the relevant population of cells encoding the relevant action, so that its activity will surpass a certain threshold. When planning time was sufficient, the onset movement time was minimal, indicating that when the visual target was presented in a timely manner, the activity was at a high enough level, only waiting for the Go cue to activate the motor activity.

Our findings differ from previous findings in that we show that the RTs do not plateau after initial decrease in RT, but rather there exists an individual planning time that allows a minimal RT, after which the RT escalates again. Hence, we ask, what can be the cause of this escalation? In the case when the Go signal is further delayed, we propose to further extend the existing models and suggest involvement of processes in the hippocampus and its connection to a neurocortical buffer with the essential modulation of the basal ganglia.

We accordingly suggest the following model: in our experiment, as the first tone is heard, populations coding movement towards four possible targets become active, in accordance with the affordance competition hypothesis (Cisek, 2007). When the selected target appears, the population coding of the movement towards the selected target passes a threshold. This process is modulated by the basal ganglia that enhance activity in motor population of movement towards the target through the direct pathway and inhibit activation in the competing neural populations through the indirect pathway (Melillo and Leisman, 2009; Friend and Kravitz, 2014). When the activity exceeds a threshold, it is gated by hippocampus oscillations active in the neocortical buffer. If the Go tone is presented within an adequate window of time, the movement plan is immediately ready to be activated. However, if additional time passes, the hippocampal–neocortical buffer oscillations start a decay process and the motor plan needs to be reactivated upon the Go cue.

The involvement of hippocampal-neocortical oscillations in linking sequential items through their subsequent maintenance has been suggested by Jensen and Lisman (2005). Specifically, Jensen’s theory posits that recently active items can be maintained in a temporally compressed buffer within the hippocampal theta oscillation such that cells representing each item can fire sequentially within the short time range of long-term potentiation (Jensen and Lisman, 2005). Moreover, recent findings have shown that patients with damage to the hippocampus have difficulties in goal-directed planning (Vikbladh et al., 2019). The basal ganglia are an important modulator of this connection is, and have been shown to play a pivotal role in movement control (Chakravarthy et al., 2010; Stocco et al., 2010). It is known the basal ganglia interact with the frontal cortex, and with the hippocampus (Alexander et al., 1991; Leisman et al., 2014). Specifically, the caudate has been shown to influence the hippocampal theta rhythm (La Grutta and Sabationo, 1988).

Another difference between the individual onset times patterns is the noisiness of the results. Some of the participants had more than one local minimum while others have a clear decrease and increase. High between- and inter-subject variability are highly linked to dopamine modulation processes. Cools and D’Esposito (2011) highlight the variability in baseline levels of DA of different individuals. Inter-subject variability may also be related to dopaminergic processes, as can be seen in ADHD, where increased RT variability is one of the most consistent neuropsychological finding in literature (Castellanos and Tannock, 2002).

To summarize, we found that RTs of healthy subjects have a characteristic U-shaped pattern. These findings should be further examined on a larger set of subjects with a longer range of planning intervals. We believe it may be beneficial to examine what is the typical pattern of individuals with neurological disorders as Parkinson’s disease and ADHD, particularly as these disorders are known to involve the basal ganglia that is an important motor modulator.

## Conclusion

We have shown that reaction time shortens as more available planning time is provided. This shortening does not plateau, but rather reaches a minimal value and then ascends again. While this U-shaped pattern was found for generally with an of average 422 ms before the Go cue, the pattern of timing had individual characteristics for each participant. We suggest that this unique pattern of dynamics supports the involvement of resonating of the motor neural population with neocortical-hippocampal oscillations.

## Ethics Statement

The protocol was approved by the Technion Institute of Science Ethics comittee. All subjects gave written informed consent in accordance with the Declaration of Helsinki.

## Author Contributions

AD programmed the experiment, ran it on subjects, performed initial data processing, and performed the relevant literature review and conclusion. RB did all the advanced data processing and statistical tests and wrote the “Results” section. MR was the Principal Investigator (P.I.) and supervisor of the experiment and took part in the ideas for the experiment and theory behind it.

## Conflict of Interest Statement

The authors declare that the research was conducted in the absence of any commercial or financial relationships that could be construed as a potential conflict of interest.
